# Effects of preoperative recombinant Interleukin 2-based immunomodulation on outcome after gastrointestinal cancer surgery: a systematic review and meta-analysis

**DOI:** 10.1038/s41416-025-03304-x

**Published:** 2026-01-17

**Authors:** A. Horcicka, N. Bewersdorf, E. Kalkum, S. Zimmermann, L. Grüßer, S. Dehne, M. A. Weigand, R. Klotz, J. Larmann

**Affiliations:** 1 Department of Anaesthesiology, Medical Faculty Heidelberg, Heidelberg University, Heidelberg, Germany; 2 Department of General, Visceral and Transplant Surgery, Medical Faculty Heidelberg, Heidelberg University, Heidelberg, Germany; 3 The Study Centre of the German Surgical Society, Heidelberg University Hospital, Heidelberg, Germany; 4 Medical Faculty Heidelberg, Institute for Medical Biometry, Heidelberg University, Heidelberg, Germany; 5 Department of Anaesthesiology, Medical Faculty, RWTH Aachen University, Aachen, Germany

**Keywords:** Surgical oncology, Gastrointestinal cancer, Tumour immunology

## Abstract

**Background:**

Patients undergoing gastrointestinal cancer surgery are often immunocompromised and susceptible to infectious complications. Recombinant Interleukin 2 activates effector immune cells and stimulates the expansion of regulatory T-cells, making it a promising intervention for prevention of inflammatory complications.

**Objective:**

Our objective was to investigate effects of different preoperative rIL2 dosages on postoperative outcome parameters.

**Methods:**

We conducted a systematic literature review and meta-analysis and included RCTs that recruited adult patients undergoing gastrointestinal cancer surgery who received preoperative subcutaneous rIL2. We performed a systematic search of MEDLINE (via PubMed), Web of Science and the Cochrane Central Register of Controlled Trials (CENTRAL) from 1989 up to April 18th, 2024.

**Results:**

Out of 2324 screened studies, we included 13 RCTs with a total of 504 patients. Lymphocyte counts [cells/mm^3^] at 1 week postoperative were higher in the intervention compared to the control group (MD 865 (95%CI: 26, 1705)). Surgical site infections and systemic infections were less likely to occur in the intervention group (OR 0.13 (95%CI: 0.03, 0.50); OR 0.25 (95%CI: 0.10, 0.66)). Severe side effects of rIL2 were not reported.

**Conclusion:**

Preoperative rIL2-based immunomodulation prevents postoperative immunosuppression while the occurrence of severe side effects does not seem to be relevant.

## Introduction

Postoperative complications are frequent and a major cause of death following surgery [1]. Patients with gastrointestinal cancer, who are often immunocompromised, are particularly susceptible to perioperative complications [2, 3]. Common complications include SSI, anastomotic leakage and systemic infections following surgical complications or associated with urinary tract infections or pneumonia [4–7]. Those complications do not just seem relevant for surgical outcome but may also impact patients’ postoperative clinical progress on the ICU floor developing severe infections with possible life-threatening complications such as sepsis. At the latest in the ICU setting the optimisation of perioperative management becomes highly relevant. According to the WHO postoperative complications also highly influence patients’ quality of life and seem to be a major global health care burden [8].

Many studies have focused on improving surgical techniques but research on identifying and optimising further modifiable risk factors in the perioperative setting is ongoing. Lately, immunomodulating concepts have been investigated not only for protection against cancer progression but also for prevention of perioperative complications [9–11].

Interleukin 2 (IL2) is a pleiotropic human cytokine that has several immunomodulating effects and is seen as a promising agent in cancer therapies [12–15].

Our objective was to investigate effects of different preoperative subcutaneous recombinant Interleukin 2 (rIL2) dosages on postoperative outcome parameters including surgery-induced immunosuppression, SSI, systemic infections, survival and side effects of rIL2 in patients undergoing gastrointestinal cancer surgery.

## Methods

This review follows the recommendations of the Cochrane Handbook for Systematic Reviews and Interventions [16]. It is in line with the preferred reporting items for systematic reviews and meta-analyses (PRISMA-statement) [17]. A review protocol was developed a priori and was submitted for registration to PROSPERO (CRD42022331437).

### Eligibility criteria

#### Participants

We included trials that recruited adult patients (age ≥ 18 years) undergoing gastrointestinal cancer surgery including colorectal, gastric, liver, oesophageal and pancreatic cancers, cancers affecting the anus, appendix, bile duct, gallbladder and small intestine, gastrointestinal neuroendocrine tumours and stromal tumours. Patients who only underwent metastatic resections or received surgical treatment of other than gastrointestinal tumours such as urological or gynaecological cancers were excluded from the study.

#### Intervention and comparator treatment

Studies were included if patients in the intervention group received preoperative rIL2. During our screening process we did not include studies in which rIL2 was primarily administered for cancer therapy. We included all dosages and lengths of application if rIL2 was applied subcutaneously. We excluded studies with intravenous or local application of rIL2.

Studies were not considered eligible if patients received additional perioperative chemoimmunotherapy simultaneously but we included studies with adjuvant chemotherapy administration. We did not specify whether studies needed to be placebo controlled or blinded. Patients in the control group needed to undergo gastrointestinal cancer surgery and did not receive rIL2.

#### Outcomes

Concerning the surgery-induced immunosuppression, we calculated the difference of lymphocyte counts [cells/mm^3^] between baseline counts and the first, second and fourth postoperative week.

We analysed postoperative complications such as systemic infections (pulmonary or urinary tract infections) and SSI following the NICE guidelines [18]. Furthermore, we evaluated anastomotic leakages (Grade A-C) or other complications such as pancreatic fistulas and digestive haemorrhages.

We also assessed oncological long-term outcomes including overall survival, progression and relapse free survival and mortality. Additional outcomes were side effects of rIL2. Based on the ‘Common Terminology Criteria for Adverse Events (CTCAE)’ of the U.S. Department of Health and Human Services we subdivided side effects which occurred after rIL2 administration in 4 categories (1: local dermal reaction; 2: mild; 3: moderate; 4: severe) [19].

#### Study types

Randomised controlled trials (RCTs) were eligible for the systematic review and meta-analysis. We excluded publications, letters or abstracts that were not peer-reviewed. We did not apply any exclusion criteria regarding study duration or study setting.

#### Search strategy

We conducted a systematic search of MEDLINE (via PubMed), Web of Science and Cochrane Central Register of Controlled Trials (CENTRAL) up to April, 18^th^ 2024 with no language restrictions. As rIL2 was officially approved as a drug in Europe in 1989 and in the United States in 1992, we searched for studies conducted since then. The search strategy was built using a combination of the following key search terms and synonyms as index or free text words and MeSH terms: Interleukin 2 (including synonyms: Interleukin II, Aldesleukin, Proleukin), gastrointestinal cancer surgery, RCT.

#### Study selection

The standard methodological procedures recommended by Cochrane were used: Two authors (AH and NB) independently and in duplicate assessed the titles and abstracts for inclusion criteria using the predetermined eligibility criteria [20]. After the first stage, both reviewers evaluated full texts and decided if the inclusion criteria were met.

Included studies were assessed for cohort overlap. Patient duplication could be excluded for most studies based on reported recruitment periods, in- and exclusion criteria (i.e. inclusion of different tumour entities) or because of differing rIL2 dosing regiments.

**Fig. 1:**
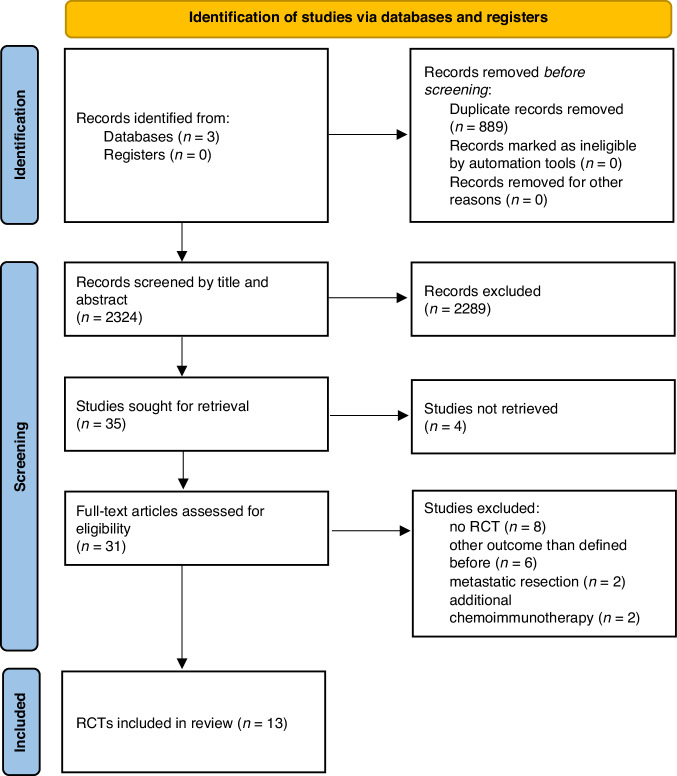
PRISMA flow diagram: Identification of studies via databases and registers. Adapted From: Page MJ et al.: The PRISMA 2020 statement: an updated guideline for reporting systematic reviews [17] n number, RCT randomised controlled trial.

Any disagreement regarding inclusion was resolved by discussion with a third member of the review team (JL). Detailed information on the screening process is illustrated in the PRISMA flow diagram (Fig. 1).

#### Data extraction

Data were extracted using a predesigned data extraction excel sheet by two review authors (AH and NB) independently and in duplicate. Inconsistencies were discussed and if necessary, a third party was consulted to reach a consensus.

In case of missing data concerning relevant information not available in the article, we contacted authors twice via email.

#### Data items

The required information included the author´s name, publication year and country, mean age and gender distribution of trial participants, type of gastrointestinal tumours of participants, rIL2 dosage, rIL2 side effects and information regarding the above-mentioned outcomes.

#### Risk of bias assessment

The methodological quality of included RCTs was assessed independently by two review authors (AH, NB) using the Cochrane Collaboration tool for assessing risk of bias 2.0 (ROB2) [21]. The tool includes five standard domains of bias: bias arising from the randomisation process, bias due to deviations from intended interventions, bias due to missing outcome data, bias in measurement of the outcome and bias in selecting of the reported results. The domains were rated as (i) high or (ii) low risk of bias, (iii) some concerns or (iv) unclear. Finally, an overall risk of bias judgement was determined which is defined as domain six in the ROB2 tool.

#### Certainty of evidence

Certainty of evidence was assessed independently by two reviewers using the ‘Grading of Recommendations, Assessment, Development and Evaluation (GRADE) approach’ [22] and divided into high, moderate, low and very low quality of evidence.

We downgraded each parameter by one level for serious concerns and by two levels for very serious concerns about risk of bias, inconsistency, indirectness, imprecision or publication bias. In the assessment of quality we included the following outcomes: lymphocyte count 1 week postoperatively, lymphocyte count 2nd week postoperatively, SSI, systemic infection, anastomotic leakage, other complications and mortality.

#### Statistical analysis

A random-effects model for pairwise meta-analysis was conducted for all outcomes where enough data was available. Petos correction method was employed which is preferable in sparse data situations like this one [23]. Since low event rates were mostly expected, Petos approach should provide relatively unbiased estimates which suffice in this exploratory setting, despite situations like these being rather challenging in general [24]. Nonetheless it should be noted that no currently available method is without drawbacks for small exploratory meta-analyses like this one.

## Results

Following removal of duplicates (*n* = 889), our database search identified 2324 records. Of these, 2289 were excluded due to ineligibility after title- and abstract screening. A total of 35 full texts were assessed. Of these, 22 were excluded because of wrong study type (*n* = 8), wrong study outcome (*n* = 6), no full-text available (*n* = 4), wrong study population (metastatic resection and no resection of the primary tumour, *n* = 2) or wrong intervention (additional chemoimmunotherapy given in the immediate perioperative period, *n* = 2) (see Fig. 1). In total, we included 13 RCTs [25–37].

Key characteristics of the identified trials are listed in Table 1.

**Table 1:** Key characteristics of the study population.

Study, Publication year	Place of Publication	Journal	Duration of trial (months)	GI tumour treated	Follow-up time (months)	Patients included (*n*)		Age, years (mean (± SD)/median (range))		Gender (%)			
						IG	CG	IG	CG	IG Male/Female		CG Male/Female	
Angelini et al. [25]	Milano, Italy	Hepato-gastroenterology	n.i	pancreatic	42	9	10	66 (43–78)	63 (35–78)	6 (67)	3 (33)	7 (70)	3 (30)
Brivio et al. [29]	Milano, Italy	Oncology	n.i	colorectal	n.i.	12	18	52.5 (35–78)	54 (34–76)	10 (83)	2 (17)	12 (67)	6 (33)
Brivio et al. [27]	Milano, Italy	Oncology	n.i	colorectal	23	24	26	59 (35–80)	57 (37–76)	13 (54)	11 (46)	14 (53)	12 (47)
Brivio et al. [28]^a^	Milano, Italy	J Biol Regul Homeost Agents	n.i	colorectal	n.i.	6	8	58 (54–67)	56 (51–66)	4 (66)	2 (33)	5 (62)	3 (38)
Brivio et al. [30]^a^	Milano, Italy	Journal of Biol. Reg. and homoeostatic agents	n.i	colorectal	n.i.	12	21	59 (48–67)	57 (49–66)	7 (58)	5 (42)	13 (62)	8 (25)
Brivio et al. [26]^a^	Milano, Italy	Anticancer Research	71	colorectal	54	43	45	64 (39–79)	65.7 (42–79)	25 (59)	17 (40)	29 (65)	15 (35)
Cesana et al. [31]	Milano, Italy	Annals of Surgical Oncology	45	gastric	51	36	32	68	69	25 (69)	11 (31)	16 (50)	16 (50)
Deehan et al. [32]	Aberdeen, Scotland	European Journal of Surgical Oncology	n.i	colorectal	10	9	9	70 (42–77)	66 (55–82)	6 (67)	3 (33)	7 (78)	2 (22)
Lissoni et al. [33]	Milano, Italy	Journal of Biological Regulation and homoeostatic agents	n.i	Colorectal, gastric, pancreatic and others	n.i.	10	10	n.i	n.i	n.i	n.i	n.i	n.i
Nichols et al. [34]	Leeds, UK	Cancer Research	n.i	colorectal	n.i.	12	13	72.7 (60–88)	72.8 (50–86)	8 (67)	4 (33)	8 (62)	5 (38)
Romano et al. [36]	Milano, Italy	Surgical Oncology	39	gastric	26	34	35	68 (42–79)	66 (48–82)	22 (61)	12 (39)	22 (63)	13 (37)
Romano et al. [35]	Milano, Italy	Hepato-gastroenterology	15	gastric	24	19	20	72 (58–79)	66 (48–82)	13 (68)	6 (32)	13 (65)	7 (35)
Uggeri et al. [37]	Milano, Italy	Hepato-gastroenterology	36	pancreatic	n.i.	9	13	65 (44–77)	63 (34–79)	6 (67)	3 (33)	8 (62)	5 (38)
Uggeri et al. [37]	Milano, Italy	Hepato-gastroenterology	36	pancreatic	n.i.	9	n.i	73 (50–80)	n.i	6 (67)	3 (33)	n.i	n.i

Studies ordered alphabetically.

*et al*. et alia, *SD* standard deviation, *n* number, *IG* intervention group, *CG* control group, *n.i.* no information.

^a^Overlap of patient cohorts cannot be fully excluded.

As Lissoni et al. allocated patients to 3 groups (rIL2 group, rIL2 + melatonin group, surgery only group) and did not give information about the age and gender separately for all three groups we could not identify the exact age and gender for the rIL2 and the control group. Data were only given for all three groups in total and we did not include the rIL2+melatonin group in our meta-analysis [33].

Uggeri et al. compared different treatment durations of rIL2 dividing the study population in three groups receiving either one, two or three days administration of rIL2. For better comparison purposes we separately looked at these groups in Table 1 [37].

Studies for which patient overlap could not be fully excluded are marked with an asterix in Table 1. However, potential overlap did not affect the results of the meta-analysis, because for all outcomes reported, a maximum of one of the respective studies contributed to the results. For the other studies in question the weight was 0%.

### Interleukin dosage

Dosage and length of application of rIL2 differed between the trials: a low dosage of 3 million IU/d was applied in one trial [34], 9 million IU/d was applied in four trials [32, 35–37]. In all other trials a high dosage of rIL2 was applied with either 12 million IU/d [26, 28, 30, 37] or 18 million IU/d [25, 29, 31, 33]. In Nichols 1992, rIL2 was applied for 5 consecutive days preoperatively whereas in all other trials rIL2 was administered for 3 consecutive days preoperatively.

In all trials surgery was performed at least 36 h after the last rIL2 application which is the time when rebound lymphocytosis occurs after s.c. rIL2 injection [29, 38].

### Risk of bias within studies

Risk of bias was assessed for ‘lymphocyte counts’, ‘long-term survival’ and ‘postoperative complications’ as those were the outcomes for which sufficient data to perform a meta-analysis was available. Postoperative complications include SSI, systemic infections, anastomotic leakages and other complications. As less than 10 studies were included in this analysis, publication bias was not feasible to be assessed with funnel plots [20].

None of the studies received a low risk of bias score on all assessed items. Consensus between the assessor’s and algorithm’s judgement was met in almost all studies assessed. However, for eight trials the algorithm’s overall judgement was high, while the assessors judged only seven studies as having a high overall risk of bias. For Angelini 2006 there was a difference in algorithm’s and assessor’s assessment regarding ‘postoperative complications’. The assessment for the outcomes ‘lymphocyte counts’ and ‘postoperative complications’ showed a difference for Brivio 2000, Brivio 2001, Brivio 1992 and Romano 2006.

Particularly the domains ‘measurement of outcome’ and ‘selection of the reported results’ resulted in ‘high risk of bias’ and ‘bias of some concerns’ for nearly all studies. Regarding the ‘randomisation process’, ‘deviations from intended interventions’ and ‘missing outcome data’ the biases were mainly assessed as ‘bias of some concerns’ and ‘low’.

None of the trials were reported to be industry funded. However, for three trials the authors report that the study drug (Aldesleukin®) was supplied by the respective manufacturers [27, 29, 30]. Also, four of the included studies are authored by a co-author affiliated with the respective manufacturer of the study drug [27, 28, 30, 33].

### Certainty of evidence

We downgraded each outcome parameter for at least two levels which means none of them was rated for high or moderate quality of evidence. Downgrading of outcome parameters of each study was especially due to high imprecision, high risk of bias and high inconsistency. For many outcome parameters we did not obtain sufficient data to detect a precise effect estimation. All outcomes are presented in Supplemental Table S1.

### Outcome parameters

Due to sparsity in the available data not every outcome parameter could be accounted for methodological and clinical differences.

### Lymphocyte counts

**Fig. 2:**
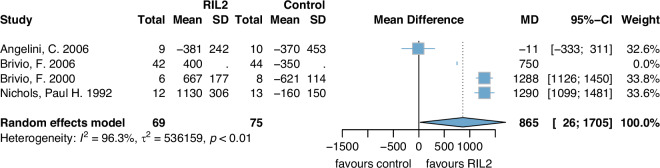
Lymphocyte count 1 week postoperatively. rIL2 recombinant Interleukin 2, SD standard deviation, MD mean difference, CI confidence interval.

Lymphocyte count [cells/mm^3^] at 1 week postoperatively was significantly higher in the rIL2 group compared to the control group (MD 865 (95%CI: 26, 1705)) (Fig. 2). Heterogeneity was high with *I*^2^ = 96%. Certainty of evidence was downgraded by three points (risk of bias, inconsistency and imprecision) until very low.

Two studies with 88 patients reported data on the lymphocyte count in the 2nd week. Lymphocyte count [cells/mm^3^] at 2 weeks postoperatively was significantly higher in the rIL2 group compared to the control group (MD 658 (95%CI: 652, 1968)) (Fig. S1).

Because of missing data, we were not able to perform a meta-analysis of the mean difference of lymphocyte count after 4 weeks compared to baseline.

### Surgical site infection (SSI)

**Fig. 3:**
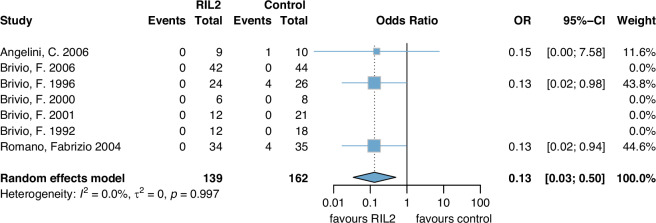
SSI (surgical site infections). rIL2 recombinant Interleukin 2, OR odds ratio, CI confidence interval.

A total of 301 patients were included of which none showed an event in the rIL2 group versus nine events that occurred in nine patients in the control group (OR 0.13 (0.03, 0.50)) (Fig. 3). Certainty of evidence was downgraded by two points (one in the category ‘risk of bias’ and one in ‘imprecision’) and assessed to be low.

### Systemic infections

Seven studies were included in the data analysis for systemic infection (such as pulmonary or urinary tract infections). Three out of seven studies reported events. There was a significant difference between the rIL2 and the control group (OR 0.25 (0.10, 0.66)) (Fig. S2).

### Anastomotic leakage

Data on anastomotic leakage was reported in seven studies comprising 301 patients. In total 10 anastomotic leakage events were documented in two studies (OR 0.68 (0.19, 2.48)). Brivio 2006 reported four anastomotic leakages occurring in the rIL2 group and five in the control group. Certainty of evidence was downgraded for one point in ‘risk of bias’ and for one point in ‘imprecision’ and assessed as low. The Forest Plot of Anastomotic leakage is exposed in Supplemental Fig. S3.

### Other complications

Only one of seven included studies reported occurrence of other complications such as pancreatic fistula and digestive haemorrhage with two events in the rIL2 group and one event in the control group [25] (see Supplemental Table S2 (Study Endpoints), OR 2 (0, 26)). We assessed very low certainty of evidence by downgrading in the following categories (each one point): risk of bias, inconsistency and imprecision. The Forest Plot of Other complications is exposed in Supplemental Fig. S4.

### Mortality

**Fig. 4:**
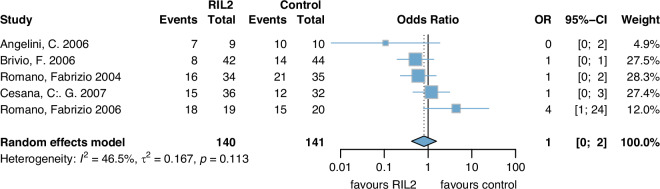
Mortality. rIL2 recombinant Interleukin 2, OR odds ratio, CI confidence interval.

Five studies were included and pooled with a total of 281 patients (Control group: 141, rIL2: 140) (Fig. 4). The follow up time differed among the trials from a median of 2 years in Romano 2004 and Romano 2006 [35, 36], 3 years in Angelini 2006 [25], 4.5 years in Brivio 2006 [26] and 6 years in Cesana 2007 [31]. No significant differences in mortality were found between the two groups (OR 1.0 (0, 2)). However, the evidence is limited due to high heterogeneity (*I*²: 46%), and the certainty of the evidence is very low because of concerns regarding risk of bias, inconsistency and imprecision.

Only four studies reported data on long-term survival. Supplemental Table S3 shows the overall survival rates after 1, 3 and 5 years in detail. No studies reported data on progression and relapse free survival.

### Side effects of rIL2

In total, 11 studies reported side effects. These were mainly category 1 and 2 side effects following the ‘Common Terminology Criteria for Adverse Events (CTCAE)’ [19] which include local dermal reactions, fever (≤39 °C) and flu-like symptoms. Two studies reported moderate (category 3) side effects (fever > 39 °C). Less side effects appeared in the control group. Overall, no severe side effects were reported (Table 2).

**Table 2:** Interleukin dosages and side effects.

Study, Publication year	IL2 dosage (million IU/day)	Local dermal reaction (*n*)	Mild reaction (*n*)	Moderate reaction (*n*)	Severe reaction (*n*)	*P* value
Angelini et al. [25]	18	9	2	0	0	<0.05
Brivio et al. [29]	18^a^	0	10	0	0	n.i
Brivio et al. [27]	18	0	13	0	0	<0.05
Brivio et al. [28]	12	0	0	0	0	n.i
Brivio et al. [30]	12	0	0	0	0	n.i
Brivio et al. [26]	12	1	30	12	0	n.i
Cesana et al. [31]	18	36	25	0	0	n.i
Deehan et al. [32]	9	2	8	0	0	n.i
Lissoni et al. [33]	18	0	4	7	0	n.i
Nichols et al. [34]	9	n.i	n.i	n.i	n.i	n.i
Romano et al. [36]	9	34	25	0	0	<0.05
Romano et al. [35]	9	0	14	0	0	<0.05
Uggeri et al. [37]	9	13	2,8	0	0	n.i
Uggeri et al. [37]	12	n.i	n.i	n.i	n.i	n.i

Studies in alphabetical order.

*IU* international units, *n* number, *et al*. et alia, *n.i*. no information.

A *p* < 0.05 states that there is a significant difference between the rIL2 and the control group in favour of the rIL2 group.

^a^18 million IU/day/m².

## Discussion

This systematic review and meta-analysis of 13 RCTs demonstrated that patients who received preoperative rIL2 had increased postoperative lymphocyte counts, fewer SSIs and less systemic infections. Though the available evidence for beneficial effects is heterogenous and risk of bias is high.

Our results suggest that preoperative rIL2 promotes immunomodulation and may counteract postoperative immunosuppression. To the best of our knowledge this is the first systematic review and meta-analysis on perioperative immunomodulation with rIL2. RIL2 is an established agent which is known to have pleiotropic effects on numerous cell lines [39]. While there was considerable interest in its use for immunomodulation in oncology, only few groups advanced the field of rIL2-based perioperative immunomodulation.

There are various reasons for postoperative immunosuppression. Surgery-induced trauma results in high levels of postoperative damage-associated molecular patterns (DAMPs) which can cause dysfunction and dysregulation in immunomodulatory cells. Certain individuals such as cancer patients are particularly susceptible to postoperative immunosuppression [40]. In an animal model Tai et al. could prove that extreme surgical stress even entails the development of metastases after surgery [41]. Our findings are in line with data from experimental studies [42–44] which show that patients’ postoperative immunosuppression is associated with downregulation of immune cells, SSIs, systemic infections like sepsis [45] and other complications. Importantly, both gastrointestinal cancer and the impact of surgery increase risk of immunosuppression [46, 47].

Immunomodulation may be a promising tool to improve perioperative care in high-risk surgery patients. Indeed, the evidence of the investigated studies shows that patients who received rIL2 had fewer postoperative infectious complications and SSIs which may result in better long-term survival rates even though - primarily due to lack of data—our work does not show any impact. As anticipated, subcutaneous rIL2 application resulted in higher lymphocyte levels in the treatment in comparison to the control group 1 week postoperatively. However, only four studies reported data on this outcome parameter. It remains unclear, why Angelini et al. did not observe any effect [25, 26, 28, 34].

Klapper et al. showed that intravenous application of rIL2 in renal cell carcinoma patients effectively modulates perioperative immune dysfunction but causes only short-term toxicity [48]. Taking into account that Klapper et al. applied rIL2 intravenously with a different dosage (720,000 IU/kg) compared to the included studies there is a lack of data on rIL2 subcutaneous application and the optimal dosing in particular.

We did not find severe side effects even in studies administering medium or high doses of rIL2 (up to 18 million IU/day). Taking into consideration our results of the certainty of evidence (GRADE) and the overall rather high risk of bias there are limitations to our meta-analysis but in only two studies [26, 33] moderate side effects were reported. Patients appeared to have local dermal reactions regularly or mild systemic reactions such as fever. In these studies high doses of rIL2 were applied. In all other studies which applied low or even a medium dosage of rIL2 only mild side effects were reported. One could conclude that a low or medium dosage of rIL2 (up to 9 million IU/day) might be the ideal dosage to receive the desired effect but have less side effects.

Postoperative complications after major abdominal cancer surgeries are common [49], yet surprisingly, they did not often occur in the included studies of our meta-analysis. It also remains unclear why almost half of the studies do not report any complications at all. The relevant expenses associated with major complications further underline the importance of minimising perioperative complications by anaesthesiologists [50].

Only a few studies reported long-term survival with different follow-up periods (range from 2 until 6 years) [25, 26, 31, 35, 36]. Even though data are sparse and we did not consider neoadjuvant chemotherapy or potential differences in adjuvant chemotherapy our meta-analysis showed no significant difference in long-term survival. In comparison to other studies where rIL2 was applied for an extended period in patients with renal cell carcinoma survival was prolonged [51]. It seems plausible for rIL2 to have a positive impact beyond the perioperative period. It might influence cancer cell proliferation and therefore the status of metastasis or cancer recurrence rates. Overall, our findings suggest that rIL2 has beneficial immunomodulating properties making it a promising agent for perioperative patient care.

Interestingly, RIL2 may also have the potential to prevent inflammatory complications such as cardio-vascular events. In an animal model IL2 mediated Regulatory T cells (Treg) expansion and prevented surgery induced progression of atherosclerotic plaques [52]. In clinical studies high Treg levels are associated with reduced perioperative cardiovascular complications [53]. As rIL2 modulates different immune cells its preoperative application may not only minimise surgical induced complications but can also have a positive impact on perioperative cardiovascular outcome which provides an outlook to the future of perioperative immunomodulation.

### Limitations

This systematic review and meta-analysis has several limitations which warrant caution interpreting the results.

First, the methodological quality of our primary studies was low. All studies were rated with a bias of some concerns or high-risk of bias. In many studies, incomplete reporting on randomisation processes and their implementation hampers an evaluation of reliability and validity. In particular, in many studies it remains unclear if the control groups were given placebos which would presumingly result in a conduction of unblinded trials.

Concerning the certainty of evidence which is, after critical appraisal, in most cases low and very low, downgrading of outcome parameters of each study is especially due to low imprecision, high risk of bias and high inconsistency. For many outcome parameters we could not withdraw sufficient patient data to detect a precise estimate of the effect. Some parameters showed a high heterogeneity and as mentioned above none of the studies received a low risk of bias score on all assessed items [54]. The high heterogeneity especially of the outcome parameter lymphocyte count at 1 week in our meta-analysis complicates the process of drawing general conclusions. Most likely this is due to the difference in study design of the included RCTs [54]. The missing and quality of blinding in these studies appeared to be poor and therefore smallers the effect size of the meta-analysis.

We could only pool three out of four studies for lymphocyte count at week one due to missing confidence intervals of Brivio, 2006. It is possible that if the confidence interval was given the I2 value and reliability would improve.

Another limitation of the systematic review is that most of the included studies took place only in Italy, some were even conducted in the same centre [55].

Despite our efforts to contact the authors, there was often limited data concerning our predefined outcome parameters. We could only pool some of the studies in our random effects models. Further, due to partial sparseness of the available data classic diagnostic tools like I² or tau² cannot be relied on to make any statements about the solidity of our fitted models. This applies for the lymphocyte count at week 1 and overall mortality. The pooled estimates should therefore be treated as summary of the current body of evidence and not be used to draw treatment decisions.

## Conclusion

This systematic review and meta-analysis suggests that preoperative administration of subcutaneous rIL2 promotes immunomodulation in gastrointestinal surgery patients without severe side effects. However, most studies were of low methodological quality, showed a high risk of bias and high heterogeneity. Further research is needed to investigate the potential of preoperative rIL2 to improve individualised perioperative patient care. To clarify the role of rIL2 for immunomodulation in gastrointestinal cancer surgery, several clinical studies that build on each other would be necessary: (i) a dose-escalating study to identify the minimum dose necessary to rapidly expand lymphocyte and regulatory T-cell counts prior to surgery, (ii) a pilot-trial with a clinical endpoint such as surgical site infections or systemic infections to assess feasibility (i.e. to evaluate exclusion and inclusion criteria, to assess drop-out rate etc.) (iii) a small explorative multicenter trial to gain preliminary data on efficacy for different clinically relevant endpoints for planning (iv) a confirmative international multicenter randomised double-blinded controlled trial powered to test the hypothesis, that rIL2 affects a clinically relevant outcome. This strategy would advance the field and close the existing knowledge gap.

## Supplementary information

Supplemental Material

## Data Availability

All data generated or analysed during this study are included in this published article (and its supplementary information files).

## References

[1] International Surgical Outcomes Study Group. Global patient outcomes after elective surgery: prospective cohort study in 27 low-, middle- and high-income countries. Br J Anaesth. 2016;117:601–9. 27799174 10.1093/bja/aew316 PMC5091334

[2] Fortun J, Martin-Davila P, Pascual J, Cervera C, Moreno A, Gavalda J, et al. Immunosuppressive therapy and infection after kidney transplantation. Transpl Infect Dis. 2010;12:397–405. 20553437 10.1111/j.1399-3062.2010.00526.x

[3] van Kooten RT, Bahadoer RR, Peeters K, Hoeksema JHL, Steyerberg EW, Hartgrink HH, et al. Preoperative risk factors for major postoperative complications after complex gastrointestinal cancer surgery: a systematic review. Eur J Surg Oncol. 2021;47:3049–58. 34340874 10.1016/j.ejso.2021.07.021

[4] Paun BC, Cassie S, MacLean AR, Dixon E, Buie WD. Postoperative complications following surgery for rectal cancer. Ann Surg. 2010;251:807–18. 20395841 10.1097/SLA.0b013e3181dae4ed

[5] Kurita N, Miyata H, Gotoh M, Shimada M, Imura S, Kimura W, et al. Risk model for distal gastrectomy when treating gastric cancer on the basis of data from 33,917 Japanese patients collected using a nationwide web-based data entry system. Ann Surg. 2015;262:295–303. 25719804 10.1097/SLA.0000000000001127

[6] Smits FJ, Verweij ME, Daamen LA, van Werkhoven CH, Goense L, Besselink MG, et al. Impact of complications after pancreatoduodenectomy on mortality, organ failure, hospital stay, and readmission: analysis of a nationwide audit. Ann Surg. 2022;275:e222–e8. 32502075 10.1097/SLA.0000000000003835

[7] Baiocchi GL, Giacopuzzi S, Reim D, Piessen G, Costa PMD, Reynolds JV, et al. Incidence and grading of complications after gastrectomy for cancer using the GASTRODATA registry: a European retrospective observational study. Ann Surg. 2020;272:807–13. 32925254 10.1097/SLA.0000000000004341

[8] WHO. Guidelines for Safe Surgery 2009: Safe Surgery Saves Lives. Geneva; 2009. 23762968

[9] Bakos O, Lawson C, Rouleau S, Tai LH. Combining surgery and immunotherapy: turning an immunosuppressive effect into a therapeutic opportunity. J Immunother Cancer. 2018;6:86. 30176921 10.1186/s40425-018-0398-7 PMC6122574

[10] Andersson R, Andersson B, Andersson E, Eckerwall G, Norden M, Tingstedt B. Immunomodulation in surgical practice. HPB (Oxf). 2006;8:116–23. 10.1080/13651820410016660 PMC2131412 18333259

[11] Adiamah A, Skorepa P, Weimann A, Lobo DN. The impact of preoperative immune modulating nutrition on outcomes in patients undergoing surgery for gastrointestinal cancer: a systematic review and meta-analysis. Ann Surg. 2019;270:247–56. 30817349 10.1097/SLA.0000000000003256

[12] Rosenberg SA. IL-2: the first effective immunotherapy for human cancer. J Immunol. 2014;192:5451–8. 24907378 10.4049/jimmunol.1490019 PMC6293462

[13] Hernandez R, Poder J, LaPorte KM, Malek TR. Engineering IL-2 for immunotherapy of autoimmunity and cancer. Nat Rev Immunol. 2022;22:614–28. 35217787 10.1038/s41577-022-00680-w

[14] Yang JC, Sherry RM, Steinberg SM, Topalian SL, Schwartzentruber DJ, Hwu P, et al. Randomized study of high-dose and low-dose interleukin-2 in patients with metastatic renal cancer. J Clin Oncol. 2003;21:3127–32. 12915604 10.1200/JCO.2003.02.122 PMC2275327

[15] Dafni U, Michielin O, Lluesma SM, Tsourti Z, Polydoropoulou V, Karlis D, et al. Efficacy of adoptive therapy with tumor-infiltrating lymphocytes and recombinant interleukin-2 in advanced cutaneous melanoma: a systematic review and meta-analysis. Ann Oncol. 2019;30:1902–13. 31566658 10.1093/annonc/mdz398

[16] Cumpston M, Li T, Page MJ, Chandler J, Welch VA, Higgins JP, et al. Updated guidance for trusted systematic reviews: a new edition of the Cochrane Handbook for Systematic Reviews of Interventions. Cochrane Database Syst Rev. 2019;10:ED000142. 31643080 10.1002/14651858.ED000142 PMC10284251

[17] Page MJ, McKenzie JE, Bossuyt PM, Boutron I, Hoffmann TC, Mulrow CD, et al. The PRISMA 2020 statement: an updated guideline for reporting systematic reviews. BMJ. 2021;372:n71. 33782057 10.1136/bmj.n71 PMC8005924

[18] National Institute for Health and Care Excellence. NICE guidance “Conditions and diseases” United Kingdom 2025 [cited 31 January 2025]. Available from: https://www.nice.org.uk/guidance/conditions-and-diseases.

[19] US Department of Health and Human Services NIoH, National Cancer Institute. Common Terminology Criteria for Adverse Events (CTCAE) Version 5. 2017, November 27. Available from: https://ctep.cancer.gov/protocolDevelopment/electronic_applications/ctc.htm#ctc_50 [cited 20th February 2025].

[20] Higgins JPT, Chandler J, Cumpston M, Li T, Page MJ, Welch VA. Cochrane Handbook for Systematic Reviews of Interventions version 6.4 (updated August 2023). Cochrane, 2023. Available from www.cochrane.org/handbook.

[21] Higgins JP, Altman DG, Gotzsche PC, Juni P, Moher D, Oxman AD, et al. The Cochrane Collaboration’s tool for assessing risk of bias in randomised trials. BMJ. 2011;343:d5928. 22008217 10.1136/bmj.d5928 PMC3196245

[22] Guyatt GH, Oxman AD, Montori V, Vist G, Kunz R, Brozek J, et al. GRADE guidelines: 5. Rating the quality of evidence-publication bias. J Clin Epidemiol. 2011;64:1277–82. 21802904 10.1016/j.jclinepi.2011.01.011

[23] Bradburn MJ, Deeks JJ, Berlin JA, Russell Localio A. Much ado about nothing: a comparison of the performance of meta-analytical methods with rare events. Stat Med. 2007;26:53–77. 16596572 10.1002/sim.2528

[24] J. Sweeting M, J. Sutton A, C. Lambert P. What to add to nothing? Use and avoidance of continuity corrections in meta-analysis of sparse data. Stat Med. 2004;23:1351–75. 15116347 10.1002/sim.1761

[25] Angelini C, Bovo G, Muselli P, Mussi C, Crippa S, Caprotti R, et al. Preoperative interleukin-2 immunotherapy in pancreatic cancer: preliminary results. Hepato Gastroenterol. 2006;53:141–4. 16506394

[26] Brivio F, Fumagalli L, Lissoni P, Nardone A, Nespoli L, Fattori L, et al. Pre-operative immunoprophylaxis with interleukin-2 may improve prognosis in radical surgery for colorectal cancer stage B-C. Anticancer Res. 2006;26:599–603. 16739327

[27] Brivio F, Lissoni P, Alderi G, Barni S, Lavorato F, Fumagalli L. Preoperative interleukin-2 subcutaneous immunotherapy may prolong the survival time in advanced colorectal cancer patients. Oncology. 1996;53:263–8. 8692528 10.1159/000227571

[28] Brivio F, Lissoni P, Gilardi R, Ferrante R, Vigore L, Curzi L, et al. Abrogation of surgery-induced decline in circulating dendritic cells by subcutaneous preoperative administration of IL-2 in operable cancer patients. J Biol Regul Homeost Agents. 2000;14:200–3. 11037053

[29] Brivio F, Lissoni P, Tisi E, Erba L, Barni S, Tancini G, et al. Effects of a preoperative therapy with interleukin-2 on surgery-induced lymphocytopenia in cancer patients. Oncology. 1992;49:215–8. 1495748 10.1159/000227041

[30] Brivio F, Lissoni P, Perego MS, Lissoni A, Fumagalli L. Abrogation of surgery-induced IL-6 hypersecretion by presurgical immunotherapy with IL-2 and its importance in the prevention of postoperative complications. J Biol Regul Homeost Agents. 2001;15:370–4. 11860226

[31] Cesana GC, Romano F, Piacentini G, Scotti M, Brenna A, Bovo G, et al. Low-dose interleukin-2 administered pre-operatively to patients with gastric cancer activates peripheral and peritumoral lymphocytes but does not affect prognosis. Ann Surg Oncol. 2007;14:1295–304. 17225981 10.1245/s10434-006-9239-x

[32] Deehan DJ, Heys SD, Ashby J, Eremin O. Interleukin-2 (IL-2) augments host cellular immune reactivity in the perioperative period in patients with malignant disease. Eur J Surg Oncol. 1995;21:16–22. 7851545 10.1016/s0748-7983(05)80061-7

[33] Lissoni P, Brivio F, Brivio O, Fumagalli L, Gramazio F, Rossi M, et al. Immune effects of preoperative immunotherapy with high-dose subcutaneous interleukin-2 versus neuroimmunotherapy with low-dose interleukin-2 plus the neurohormone melatonin in gastrointestinal tract tumor patients. J Biol Regul Homeost Agents. 1995;9:31–3. 8553906

[34] Nichols PH, Ramsden CW, Ward U, Sedman PC, Primrose JN. Perioperative immunotherapy with recombinant interleukin 2 in patients undergoing surgery for colorectal cancer. Cancer Res. 1992;52:5765–9. 1394200

[35] Romano F, Cesana G, Caprotti R, Bovo G, Uggeri F, Piacentini MG, et al. Preoperative IL-2 immunotherapy enhances tumor infiltrating lymphocytes (TILs) in gastric cancer patients. Hepato Gastroenterol. 2006;53:634–8. 16995478

[36] Romano F, Cesana G, Berselli M, Gaia Piacentini M, Caprotti R, Bovo G, et al. Biological, histological, and clinical impact of preoperative IL-2 administration in radically operable gastric cancer patients. J Surg Oncol. 2004;88:240–7. 15565596 10.1002/jso.20155

[37] Uggeri F, Caprotti R, De Grate L, Crippa S, Nobili C, Penati C, et al. Short-term preoperative IL-2 immunotherapy in operable pancreatic cancer: a randomized study. Hepato Gastroenterol. 2009;56:861–5. 19621718

[38] Konrad MWHG, Hersh EM, Mansell PW, Mertelsmann R, Kolitz JE, Bradley EC. Pharmacokinetics of recombinant interleukin 2 in humans. Cancer Res. 1990;50:2009–17. 2317789

[39] Gaffen SL, Liu KD. Overview of interleukin-2 function, production and clinical applications. Cytokine. 2004;28:109–23. 15473953 10.1016/j.cyto.2004.06.010

[40] Tang F, Tie Y, Tu C, Wei X. Surgical trauma-induced immunosuppression in cancer: recent advances and the potential therapies. Clin Transl Med. 2020;10:199–223. 32508035 10.1002/ctm2.24 PMC7240866

[41] Tai LH, Tanese de Souza C, Sahi S, Zhang J, Alkayyal AA, Ananth AA, et al. A mouse tumor model of surgical stress to explore the mechanisms of postoperative immunosuppression and evaluate novel perioperative immunotherapies. J Vis Exp. 2014:e51253. 10.3791/51253. 10.3791/51253 PMC4146339 24686980

[42] Chiarello MM, Fransvea P, Cariati M, Adams NJ, Bianchi V, Brisinda G. Anastomotic leakage in colorectal cancer surgery. Surg Oncol. 2022;40:101708. 35092916 10.1016/j.suronc.2022.101708

[43] Tsujimoto H, Kobayashi M, Sugasawa H, Ono S, Kishi Y, Ueno H. Potential mechanisms of tumor progression associated with postoperative infectious complications. Cancer Metastasis Rev. 2021;40:285–96. 33389285 10.1007/s10555-020-09945-z

[44] Coccolini F, Improta M, Cicuttin E, Catena F, Sartelli M, Bova R, et al. Surgical site infection prevention and management in immunocompromised patients: a systematic review of the literature. World J Emerg Surg. 2021;16:33. 34112231 10.1186/s13017-021-00375-y PMC8194010

[45] Stevens NE, Chapman MJ, Fraser CK, Kuchel TR, Hayball JD, Diener KR. Therapeutic targeting of HMGB1 during experimental sepsis modulates the inflammatory cytokine profile to one associated with improved clinical outcomes. Sci Rep. 2017;7:5850. 28724977 10.1038/s41598-017-06205-z PMC5517568

[46] Zhang Y, Rajput A, Jin N, Wang J. Mechanisms of immunosuppression in colorectal cancer. Cancers. 2020;12:3850. 33419310 10.3390/cancers12123850 PMC7766388

[47] Bezu L, Akçal Öksüz D, Bell M, Buggy D, Diaz-Cambronero O, Enlund M, et al. Perioperative immunosuppressive factors during cancer surgery: an updated review. Cancers. 2024;16:2304. 39001366 10.3390/cancers16132304 PMC11240822

[48] Klapper JA, Downey SG, Smith FO, Yang JC, Hughes MS, Kammula US, et al. High-dose interleukin-2 for the treatment of metastatic renal cell carcinoma : a retrospective analysis of response and survival in patients treated in the surgery branch at the National Cancer Institute between 1986 and 2006. Cancer. 2008;113:293–301. 18457330 10.1002/cncr.23552 PMC3486432

[49] Kazaure HS, Roman SA, Sosa JA. Association of postdischarge complications with reoperation and mortality in general surgery. Arch Surg. 2012;147:1000–7. 23165614 10.1001/2013.jamasurg.114

[50] Selby LV, Gennarelli RL, Schnorr GC, Solomon SB, Schattner MA, Elkin EB, et al. Association of hospital costs with complications following total gastrectomy for gastric adenocarcinoma. JAMA Surg. 2017;152:953–8. 28658485 10.1001/jamasurg.2017.1718 PMC5710284

[51] Klatte T, Ittenson A, Rohl FW, Ecke M, Allhoff EP, Bohm M. Perioperative immunomodulation with interleukin-2 in patients with renal cell carcinoma: results of a controlled phase II trial. Br J Cancer. 2006;95:1167–73. 17031403 10.1038/sj.bjc.6603391 PMC2360567

[52] Handke J, Kummer L, Weigand MA, Larmann J. Modulation of peripheral CD4(+)CD25(+)Foxp3(+) regulatory T cells ameliorates surgical stress-induced atherosclerotic plaque progression in ApoE-deficient mice. Front Cardiovasc Med. 2021;8:682458. 34485396 10.3389/fcvm.2021.682458 PMC8416168

[53] Scholz AS, Handke J, Gillmann HJ, Zhang Q, Dehne S, Janssen H, et al. Frontline Science: low regulatory T cells predict perioperative major adverse cardiovascular and cerebrovascular events after noncardiac surgery. J Leukoc Biol. 2020;107:717–30. 31523852 10.1002/JLB.5HI1018-392RR

[54] Davey Smith G, Egger M, Phillips AN. Meta-analysis. Beyond the grand mean? BMJ. 1997;315:1610–4. 9437284 10.1136/bmj.315.7122.1610 PMC2127994

[55] Schulz KF, Chalmers I, Hayes RJ, Altman DG. Empirical evidence of bias. Dimensions of methodological quality associated with estimates of treatment effects in controlled trials. JAMA. 1995;273:408–12. 7823387 10.1001/jama.273.5.408

