# Supplementary material for: Effects of preoperative recombinant Interleukin 2-based immunomodulation on outcome after gastrointestinal cancer surgery: a systematic review and meta-analysis
Source: Br J Cancer. 2026 Jan 17;134(7):1057–65. doi: 10.1038/s41416-025-03304-x (PMC12996586; doi:10.1038/s41416-025-03304-x)
Supplement: Supplementary file 1 — Supplemental Material [file 41416_2025_3304_MOESM1_ESM.docx]

**Appendix A: Supplemental material**

**Table S1:** Certainty of Evidence, GRADE (Grading of Recommendations, Assessment, Development and Evaluation) approach

| **Outcome** | **Certainty of Evidence** |
| --- | --- |
| Lymphocyte count one week postoperatively | very low |
| Lymphocyte count 2^nd^ week postoperatively | very low |
| Surgical site infection | low |
| Systemic infection | very low |
| Anastomotic leakage | low |
| Other complications | very low |
| Survival/Mortality | very low |

**Table S2:** Study endpoints

| **Study, publication year** | Systemic infections | | Surgical site infection | | Anastomotic leakage | | Cardiovascular complications | | Other complications | |
| --- | --- | --- | --- | --- | --- | --- | --- | --- | --- | --- |
|  | IG | CG | IG | CG | IG | CG | IG | CG | IG | CG |
| **Angelini et al., 2006** | 1 | 6 | 0 | 1 | 0 | 0 | 0 | 0 | 2 | 0 |
| **Brivio et al., 1992** | 0 | 0 | 0 | 0 | 0 | 0 | 0 | 0 | 0 | 0 |
| **Brivio et al., 1996** | 1 | 4 | 0 | 4 | 0 | 0 | 0 | 0 | 0 | 0 |
| **Brivio et al., 2000** | 0 | 0 | 0 | 0 | 0 | 0 | 0 | 0 | 0 | 0 |
| **Brivio et al., 2001** | 0 | 0 | 0 | 0 | 0 | 0 | 0 | 0 | 0 | 0 |
| **Brivio et al., 2006** | 0 | 0 | 0 | 0 | 4 | 5 | 0 | 0 | 0 | 0 |
| **Cesana et al., 2007** | n.i. | n.i. | n.i. | n.i. | n.i. | n.i. | n.i. | n.i. | n.i. | n.i. |
| **Deehan et al., 1995** | n.i. | n.i. | n.i. | n.i. | n.i. | n.i. | n.i. | n.i. | n.i. | n.i. |
| **Lissoni et al., 1995** | n.i. | n.i. | n.i. | n.i. | n.i. | n.i. | n.i. | n.i. | n.i. | n.i. |
| **Nichols et al., 1992** | n.i. | n.i. | n.i. | n.i. | n.i. | n.i. | n.i. | n.i. | n.i. | n.i. |
| **Romano et al., 2004** | 2 | 6 | 0 | 4 | 0 | 1 | 0 | 0 | 0 | 0 |
| **Romano et al., 2006** | n.i. | n.i. | n.i. | n.i. | n.i. | n.i. | n.i. | n.i. | n.i. | n.i. |
| **Uggeri et al., 2009** | n.i. | n.i. | n.i. | n.i. | n.i. | n.i. | n.i. | n.i. | n.i. | n.i. |
| **Uggeri et al., 2009** | n.i. | n.i. | n.i. | n.i. | n.i. | n.i. | n.i. | n.i. | n.i. | n.i. |

IG: intervention group, CG: control group, et al.: et alia, n.i.: no information; studies ordered alphabetically

**Table S3:** Long-term survival

| **Study, Publication year** | 1-year overall survival (%) | | 3-year overall survival (%) | | 5-year overall survival (%) | |
| --- | --- | --- | --- | --- | --- | --- |
|  | IG | CG | IG | CG | IG | CG |
| **Angelini et al., 2006** | n.i. | n.i. | 22 | 0 | n.i. | n.i. |
| **Brivio et al., 1992** | n.i. | n.i. | n.i. | n.i. | n.i. | n.i. |
| **Brivio et al., 1996** | n.i. | n.i. | n.i. | n.i. | n.i. | n.i. |
| **Brivio et al., 2000** | n.i. | n.i. | n.i. | n.i. | n.i. | n.i. |
| **Brivio et al., 2001** | n.i. | n.i. | n.i. | n.i. | n.i. | n.i. |
| **Brivio et al., 2006** | n.i. | n.i. | n.i. | n.i. | 81 | 68 |
| **Cesana et al., 2007** | n.i. | n.i. | 58 | 62.5 | n.i. | n.i. |
| **Deehan et al., 1995** | n.i. | n.i. | n.i. | n.i. | n.i. | n.i. |
| **Lissoni et al., 1995** | n.i. | n.i. | n.i. | n.i. | n.i. | n.i. |
| **Nichols et al., 1992** | n.i. | n.i. | n.i. | n.i. | n.i. | n.i. |
| **Romano et al., 2004** | 75 | 52 | 53 | 40 | n.i. | n.i. |
| **Romano et al., 2006** | n.i. | n.i. | n.i. | n.i. | n.i. | n.i. |
| **Uggeri et al., 2009** | n.i. | n.i. | n.i. | n.i. | n.i. | n.i. |
| **Uggeri et al., 2009** | n.i. | n.i. | n.i. | n.i. | n.i. | n.i. |

IG: intervention group, CG: control group, et al.: et alia, n.i.: no information; studies ordered alphabetically

**Figure S1:** Lymphocyte count [cells/mm^3^] at 2^nd^ week postoperatively


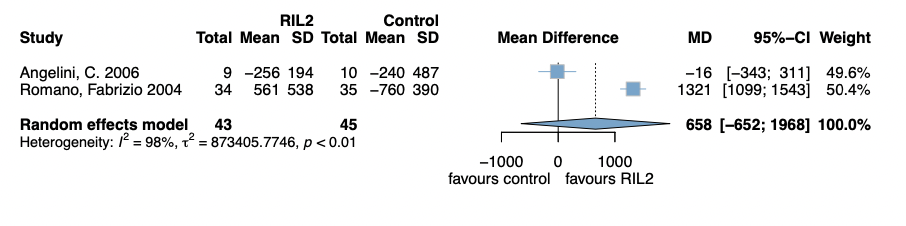


rIL2: recombinant Interleukin 2, SD: standard deviation, MD: mean difference, CI: confidence interval

**Figure S2:** Systemic infections


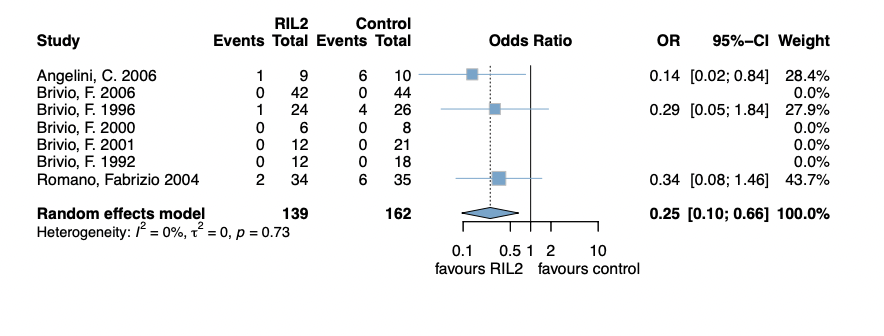


rIL2: recombinant Interleukin 2, OR: odds ratio, CI: confidence interval

**Figure S3:** Anastomotic leakage


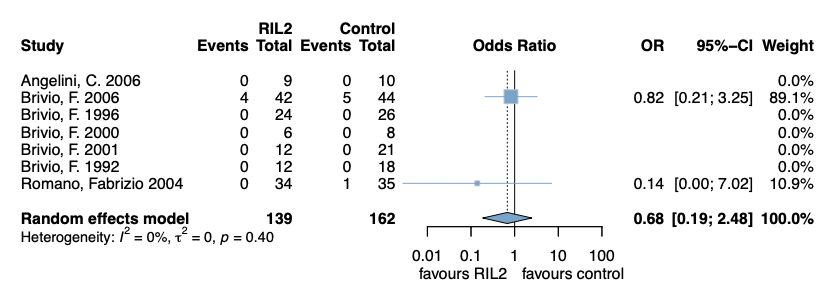


rIL2: recombinant Interleukin 2, OR: odds ratio, CI: confidence interval

**Figure S4:** Other complications


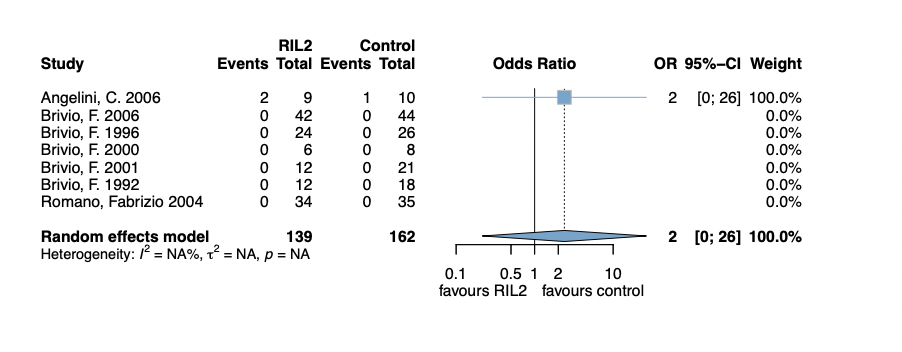


rIL2: recombinant Interleukin 2, OR: odds ratio, CI: confidence interval
